# Global Analysis of Cereal microProteins Suggests Diverse Roles in Crop Development and Environmental Adaptation

**DOI:** 10.1534/g3.120.400794

**Published:** 2020-08-06

**Authors:** Kaushal Kumar Bhati, Valdeko Kruusvee, Daniel Straub, Anil Kumar Nalini Chandran, Ki-Hong Jung, Stephan Wenkel

**Affiliations:** * Copenhagen Plant Science Centre, University of Copenhagen, Thorvaldsensvej 40, 1871 Frederiksberg C, Denmark; † Department of Plant and Environmental Sciences, University of Copenhagen, Thorvaldsensvej 40, 1871 Frederiksberg C, Denmark; ‡ Louvain Institute of Biomolecular Science, UCLouvain, 1348, Louvain-la-Neuve, Belgium; § Graduate School of Biotechnology & Crop Biotech Institute, Kyung Hee University, Yongin 446-701, Republic of Korea; ** NovoCrops Centre, University of Copenhagen, Thorvaldsensvej 40, 1871 Frederiksberg C, Denmark

**Keywords:** microProteins, protein-protein interaction, miPFinder, biotechnology, crops

## Abstract

MicroProteins are a class of small single-domain proteins that post-translationally regulate larger multidomain proteins from which they evolved or which they relate to. They disrupt the normal function of their targets by forming microProtein-target heterodimers through compatible protein-protein interaction (PPI) domains. Recent studies confirm the significance of microProteins in the fine-tuning of plant developmental processes such as shoot apical meristem maintenance and flowering time regulation. While there are a number of well-characterized microProteins in *Arabidopsis thaliana*, studies from more complex plant genomes are still missing. We have previously developed miPFinder, a software for identifying microProteins from annotated genomes. Here we present an improved version where we have updated the algorithm to increase its accuracy and speed, and used it to analyze five cereal crop genomes – wheat, rice, barley, maize and sorghum. We found 20,064 potential microProteins from a total of 258,029 proteins in these five organisms, of which approximately 2000 are high-confidence, *i.e.*, likely to function as actual microProteins. Gene ontology analysis of these 2000 microProtein candidates revealed their roles in stress, light and growth responses, hormone signaling and transcriptional regulation. Using a recently developed rice gene co-expression database, we analyzed 347 potential rice microProteins that are also conserved in other cereal crops and found over 50 of these rice microProteins to be co-regulated with their identified interaction partners. Overall, our study reveals a rich source of biotechnologically interesting small proteins that regulate fundamental plant processes such a growth and stress response that could be utilized in crop bioengineering.

MicroProteins are a class of small post-translational regulators found in both plant and animal genomes. Their defining characteristics are the presence of only a single protein-protein interaction (PPI) domain and thus small size, and their evolutionary relationship to their targets. MicroProteins (miPs) are classified into two major categories: *cis*-microProteins, which are microProteins derived from the same gene as their target protein, arise through processes such as alternative splicing, proteolytic cleavage, or alternative transcription start sites while *trans*-microProteins are paralogous to their targets, arising from genes which have undergone duplication and subsequent evolutionary trimming through domain loss (Figure 1A) (Bhati *et al.* 2018; Eguen *et al.* 2015). According to the current model, both *cis*- and *trans*-miPs function by forming heterodimers with their target proteins, thereby disrupting the normal biological function of the target homodimers (Figure 1B) (Eguen *et al.* 2015). This can result in a change in the nuclear localization (Hong *et al.* 2011), loss of protein or DNA-binding activity (Wenkel *et al.* 2007), or even the acquisition of new functionality for the miP-heterodimer pair (Figure 1C) (Graeff *et al.* 2016).

**Figure 1:**
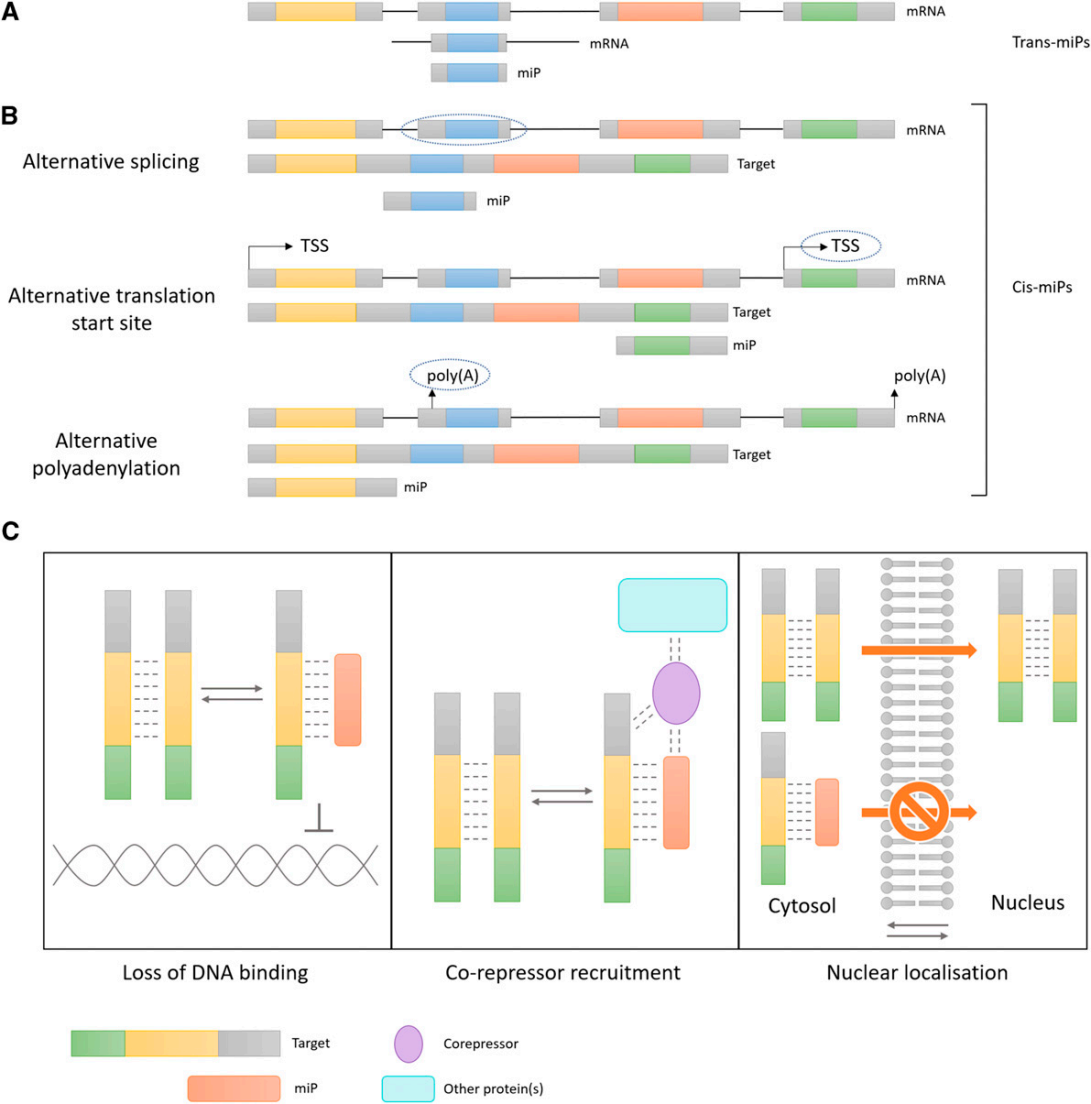
A) *Trans*-miPs are generated through the duplication of the ancestral gene (top) and subsequent evolutionary trimming (middle), leaving only the domain responsible for dimerization (bottom, blue). Black lines are introns while colored and gray boxes represent exons. B) *Cis*-miPs are derived from the same mRNA transcript (top) as their target proteins (middle) through alternative splicing, alternative translation start site or alternative polyadenylation. Alternative splicing can lead to the generation of microProteins (bottom) by only including the exon containing the dimerization domain (blue). Alternative translation start site (circled) within the mRNA of the parent protein (top) can lead to the generation of a truncated construct that encodes for the dimerization domain (bottom, green). In some instances, an alternative polyadenylation signal (circled) can lead to the generation of a shorter mRNA construct from which the protein containing the dimerization domain (bottom, yellow) is made from. TSS – Translation start site. Poly (A) – polyadenylation site. C) The balance between the target homodimers and the target-microProtein (red) heterodimers can affect many molecular functions such as DNA-binding, recruitment of a co-repressor (purple) and other accessory proteins (teal) or even nuclear localization of the target protein.

The majority of known microProteins regulate transcription factors, although recent synthetic approaches have shown that they can target proteins from diverse families (Dolde *et al.* 2018; Eguen *et al.* 2020). This has raised the prospect that microProteins could be involved in a wide range of yet unexplored physiological processes such as metabolism or stress response. In addition, there is emerging evidence that microProteins regulate biotechnologically and economically important functions such as flower development (Xu *et al.* 2019) and apical meristem maintenance (Wenkel *et al.* 2007; Kim *et al.* 2008). As such, both natural and synthetic microProteins could be utilized as potent biotechnological tools in, for example, crop bioengineering. However, while the rapid development of next-generation sequencing has given us access to a wealth of genomic data from which novel microProteins could be detected, accurate *in silico* identification of candidate microProteins remains a challenge.

We have previously developed a tool called miPFinder to find potential microProteins from annotated genomes (Straub and Wenkel 2017), which we have used to find and study microProteins in plant genomes. Here we present an updated version of the algorithm with improved accuracy that is also able to process poorly annotated genomes. Furthermore, we demonstrate that the new scoring algorithm is able to find known microProteins with high accuracy. We have used the improved miPFinder (miPFinder v2.0) to study five monocot species, namely barley, wheat, sorghum, rice and maize, and found that these genomes are a rich source of potential microProteins. We identified 20,064 potential microProtein candidates from a total of 53,469 small proteins, of which approximately 10% were predicted to be high-confidence, *i.e.*, likely to function as a microProtein. We performed gene ontology analysis on these 10% and found that many of the targets are involved in transcriptional control and stress response. Using rice as an example, we extracted all rice microProteins and their targets that are conserved in at least three other species and analyzed their biological role using the Rice Genome Annotation Project database (Kawahara *et al.* 2013). We found that the targets of these conserved microProteins are involved in regulating growth and stress response, as well as reproductive processes such as heading date and flowering time. Additionally, we analyzed the co-expression of these identified microProtein-target pairs and found 56 that showed significant positive or negative correlation, indicating that they are co-regulated and thus likely to function together within the tissues. Together, these findings will pave way for the study of novel microProteins that are possible biotechnological targets for future crop improvement such as stress tolerance or biomass production.

## Materials and methods

### Initial proteome filtering and clustering of potential microProteins

The first step of the process is to exclude any annotated proteins which have a protein existence level (as defined by UniProt) of 5. This annotation level describes proteins whose existence is uncertain and therefore may not encode for actual proteins. The proteome is divided into two parts, one containing all the potential microProteins (called candidate microProteins, cmiPs), and all the potential targets. The user can specify the division boundary, however by default miPFinder v2.0 defines proteins no larger than 150 amino acids to be cmiPs, and the rest as targets. Next all cmiPs are compared against each other using phmmer (0.1 gap open/extension penalty, BLOSUM50 scoring matrix) to find groups of homologous cmiPs. All phmmer results with a bitscore less than 30.0 are discarded as potential false positives. All cmiPs are subsequently divided into two groups, those with no identified homologs (single-copy cmiPs) and the rest (homologous cmiPs).

Each single-copy cmiP is compared against the targets using phmmer (0.1 gap open/extension penalty, BLOSUM50 scoring matrix). Equally, for each homologous cmiP, all identified homologs are aligned using Clustal Omega (version 1.2.4) with default settings to create multiple sequence alignments (MSAs). From these MSAs HMMER profiles are created using hmmbuild (version 3.1b2) and searched against the targets. All targets with a bit score lower than 10 or higher than 120 are discarded and the top 10 hits are kept. The targets are compared against the InterPro database and any target with a single annotated domain is removed. Finally, all potential microProtein candidates with more than 10 homologous microProteins are excluded.

### Scoring of potential microProteins

For each cmiP, all identified targets are individually scored according to the following formula:

$$Score=\left(\frac{1}{bitscore^{coverage}}*bitscore*weight\right)*\frac{1}{1+e^{-0.1*\left(MicroProtein\;Instability\;Index-70.0\right)}}$$

where *coverage* is defined as $\frac{cMIP\;length}{Identified\;ancestor\;length}$ and *weight* is defined as $\frac{10}{1+e^{-k*coverage*100-m}}$ where k = 0.1 and m = 50, and MicroProtein Instability Index is the calculated protein instability score (Guruprasad, Reddy, and Pandit 1990).

### Detection and analysis of crop microProteins

We downloaded the proteomes of barley (UP000011116), rice (UP000059680), wheat (UP000019116), maize (UP000007305) and sorghum (UP000000768) from UniProt. Additionally, we downloaded the Gene Ontology and InterPro annotations for each protein within these genomes from UniProt, as well as a list mapping InterPro identifiers to their entry types (*e.g.*, family, domain), and the Core ontology file from geneontology.org that maps GO identifiers to their annotated information. We used these files as an input for the miPFinder algorithm and analyzed each crop species separately. We took the top 10% of the microProtein-target pairs for each species and extracted the target GO identifiers using a Python script. The GO identifiers from each organism were first analyzed separately using agriGO v2.0 with default settings (Tian *et al.* 2017). The results from agriGO were combined into a list and analyzed using reviGO (Supek *et al.* 2011) using default similarity scoring algorithm with an allowed similarity of 0.5.

### Expression analysis for microProtein-target expression

For the co-expression analysis of rice microProteins and their targets we first converted microProteins and their targets groups into pairwise form. For each microProtein-target pair, we extracted the anatomical expression pattern across 10 different rice tissue samples using RNASeq anatomy database with help of Python script. For RNASeq data, we mapped reads to reference genome using Hisat2 aligner and estimated read count using featureCounts tool. This pairwise expression dataset was then used to calculate a Pearson correlation coefficient (PCC) in R (Chandran *et al.*, 2019). The significant correlation was selected when -0.7< r >0.7 and p-value< 0.01. The previously characterized information was mapped to the microProtein-target pair based on the locus identifier from the funRiceGenes database (Yao *et al.* 2018).

### Data availability

The open access sources for tools and data used in this study has been listed and cited at appropriate parts of manuscript (Materials and Methods, and Supplementary Information). The code for miPFinder v2.0 is available on https://github.com/ku-mip/mipfinder2. The data and script used in co-expression analysis is available from https://github.com/csg-khu/miPs. Supplemental material available at figshare: https://doi.org/10.25387/g3.12344138.

## Results

### New miPFinder algorithm uses an orthogonal approach to detect microProteins from genomes

The original miPFinder pipeline used annotated genomes in conjunction with (optional) databases such as STRING (Szklarczyk *et al.* 2019), and iPFAM (Finn *et al.* 2014) to detect microProteins. However, we found that while this approach worked for well-studied organisms for which high-quality experimental data are available, it underperformed for organisms where the genome annotation was either mostly unavailable or of poor quality. In order to ensure that the software can be reliably used on any genome, whether well-curated or computationally-annotated, we rewrote the algorithm to enrich for microProteins focusing on the primary sequence information content of the small proteins, while reusing some concepts from the previous algorithm (Straub and Wenkel 2017). We first defined a set of rules that define a potential microProtein and their interaction partners based on the characteristics of known microProtein-partner pairs (Table 1). These are well-defined traits that a potential microProtein or the target must have in order to be considered a microProtein (Straub and Wenkel 2017).

**Table 1:** Characteristics of potential microProteins and their targets used in the miPFinder v2.0 algorithm

Potential microProtein characteristics	Potential target characteristics
Homologous to their interaction partner	Homologous to the potential microProtein
Contains a single domain	Contains two or more domains
High predicted instability index	

Because the potential microProtein and target characteristics have a significant overlap with the rest of the proteome, the initial filtering steps would also include many proteins that are not real microProteins. In order to overcome this, we proceeded to develop a list of filters to further narrow down the set of identified putative microProteins and their interaction partners (Table 2, see Materials and Method for further information). These traits are associated with known microProtein-target pairs, but the exact parameter cut-off values can easily be modified by the user depending on the characteristics of the analyzed proteome. For example, in large genomes that have undergone many gene duplications, it may be necessary to increase the number of allowed homologs in order to not exclude all but the smallest protein families as the duplication of genes will increase the average protein family size. We set the criteria for small proteins to be less or equal than 150 amino acids as these should be small enough to only contain a single domain. Furthermore, we filtered out all proteins with an existence level of 5 (UniProt definition) as these may not represent actual existing proteins within the cells. We kept all microProtein homologs with a bit score between 30 and 120 as we found both too dissimilar and too similar hits reduce the accuracy of the algorithm. Finally, we removed any identified target that is less than 40 amino acids longer than the identified microProtein, as these proteins are unlikely to contain another domain due to the small size difference and are unable to act as a target.

**Table 2:** List of filters used by the miPFinder v2.0 algorithm

MicroProtein filter	Target filter
Length <=150 amino acids	Length > 150 amino acids
Has no more than 10 homologs in the proteome	No identified target has a bit score lower than 10 and higher than 120
Existence level <= 4 (as defined by UniProt)	Existence level <= 4 (as defined by UniProt)
Has an identified homologous target with a bit score of more than 30	Longer than the identified microProtein by at least 40 amino acids

While the algorithm informs the users of potential microProteins that will have to be further verified *in vitro* and *in vivo*, we wanted to ensure that the algorithm is able to correctly identify known microProteins. To this end, we used a set of 23 known microProteins from *Arabidopsis thaliana* (Table 3, Supplementary Table 1) and analyzed the results after applying each filtering step to make sure that they do not lead to an increase in false negatives. Additionally, we examined the results to ensure that proteins deemed unlikely to be microProteins based on their known function were removed. These included, for example, small highly conserved single-domain proteins that are part of large protein families such as calmodulins and thioredoxins. These steps also helped reduce the erroneous identification of microProtein-target pairs for large families of small proteins where some members happened to be larger than the microProtein size cut-off, leading them to be mistakenly identified as a highly conserved targets of the smaller family members. Finally, due to the relatively small size of the reviewed Arabidopsis proteome (15877 proteins), we changed the maximum allowed homologs to 8 as this made the algorithm perform better as indicated by the relative ranking of the known microProteins as well as by manual inspection of the final results. Taken together, these filtering steps enriched for the presence of known microProteins in *A. thaliana* while reducing the number of presumed contaminants.

**Table 3:** A list of known A. thaliana microProteins scored using the miPFinder v2.0 algorithm

MicroProtein UniProt ID	TAIR ID	Ranking (percentile)
Q9M157	AT4G01060	1 (1)
Q9LJW5	AT3G28917	2 (1)
O22059	AT2G46410	3 (1)
D3GKW6	AT2G30432	4 (1)
Q9LNI5	AT1G01380	6 (2)
Q8GV05	AT5G53200	7 (2)
B3H4X8	AT2G30424	10 (3)
Q84RD1	AT2G30420	14 (4)
Q1G3I2	AT4G15248	16 (4)
Q9CA51	AT1G74660	18 (5)
Q2Q493	AT1G18835	19 (5)
Q9LRM4	AT3G21890	20 (5)
Q9SJH0	AT2G42870	49 (12)
Q9FLE9	AT5G39860	52 (13)
Q8GW32	AT1G26945	55 (14)
Q9LXG5	AT5G15160	59 (15)
F4HXU3	AT1G14760	61 (15)
Q9CA64	AT1G74500	63 (15)
Q9LXR7	AT3G58850	68 (17)
Q9LJX1	AT3G28857	69 (17)
F4JCN9	AT3G47710	88 (21)
Q9LXI8	AT3G52770	189 (45)
Q56WL5	AT2G36307	191 (46)

### miPFinder v2.0 detects known microProteins and their targets with high accuracy

While the initial filtering steps enriched for potential microProtein candidates based on the *A. thaliana* dataset, the resulting list of potential microProteins (420) from Arabidopsis was too large for in-depth characterization. We wanted to assign a score to each potential microProtein based on the likelihood that the identified candidate was a real microProtein. Using a number of different (including biochemical) properties of *A. thaliana* known microProteins, we created a scoring algorithm which takes into account a number of different factors such as the length of the microProtein and the length of the potential interaction partner. In addition, the algorithm considers the likelihood of the interaction partner being a homolog of a given potential microProtein, as well as the predicted instability index (see Materials and Methods for further information).

The new scoring algorithm performs very well, ranking the majority (21/23) of known microProteins in the top 20% percentile of the results (Table 3). Only two proteins, Q9LXI8 (called LITTLE ZIPPER 3) and Q56WL5 (LITTLE ZIPPER 4) proteins, are found much lower in the results list. While these LITTLE ZIPPER protein family members have been experimentally shown to interact with class III homeodomain leucine-zipper (HD-ZIPIII) transcription factors from which they evolved (Wenkel *et al.* 2007; Floyd *et al.* 2014), they show very weak homology to their known targets. As one of the most important aspects of scoring is the identification of high-confidence targets, this inevitably meant that they would be ranked much lower than other microProteins which share high homology with their known targets. Nevertheless, the algorithm correctly identifies them as potential microProteins, and ranks them in the top half of the final results list, demonstrating that it is able to deal with cryptic microProtein candidates.

We also looked at the targets of the known microProteins as predicted by STRING (Szklarczyk *et al.* 2019) and BioGrid (Stark *et al.* 2006) databases. While not all microProtein-target interactions have been extensively verified, we found that miPFinder is able to correctly predict well-characterized targets. For example, both Q9LXI8 (LITTLE ZIPPER 3) and Q56WL5 (LITTLE ZIPPER 4) have the HD-ZIPIII members correctly identified as interacting partners (Wenkel *et al.* 2007). Equally, Q1G3I2 and Q9LRM4 both have their actual target CONSTANS predicted as an interactor by miPFinder (Graeff *et al.* 2016). For others, the predicted targets were close homologs of the true interactor. For example, Q9M157 had GL3 as a predicted interactor instead of GL1, while O22059 had predicted targets of the MYB transcription factor family, but none of them matched the true target MYB66. On the other hand, there were also microProteins whose predicted targets did not match those identified by the databases, such as Q9FLE9 and Q9SJH0. However, it is possible that the target predicted by the miPFinder is the physiological one, as these databases incorporate both experimental and *in silico* predictions such as text mining (*e.g.*, STRING) and therefore the absence of miPFinder-identified targets does not necessarily indicate a false discovery, although we did not test this experimentally. Taken together, our analysis shows that the new miPFinder program is able to both accurately detect microProteins from a genome, and correctly identify the targets (or closely related homologs) of known microProteins.

### Crop genomes are a rich source of microProteins

We wanted to test our new algorithm and explore the potential microProteins in commercially important cereal crops which could be used as a novel bioengineering targets. We analyzed five monocotyledonous cereal proteomes (wheat, barley, maize, sorghum, rice) using miPFinder, and found a moderate enrichment of potential microProteins in these organisms (Table 4, Supplementary Figure 1A, B; Supplementary Table 2). There was a large variation present in the fraction of detected small proteins and microProteins in these proteomes. For example, 33% of the rice proteome and 27% of the barley proteome consists of small proteins, compared to only 14% and 19% in wheat and maize respectively. In wheat 30% of all small proteins were predicted to be microProteins compared to 52% in maize. We also checked whether the microProteins have identifiable homologs in the genome and found no clear relationship between the proteome size and the fraction of single-copy *vs.* homologous candidate microProteins (Table 4). Both barley and maize had a much higher proportion of homologous microProteins, while rice and sorghum contained more single-copy microProteins. The only exception was wheat which displayed a much higher proportion of homologous candidate microProteins (84%) than other organisms. A possible reason for this is that the wheat genome is hexaploid, having formed through hybridization of three species. As such, there are likely to be many more homologous (technically homeologous) genes (functionally alleles) than in the other diploid monocots that we analyzed.

**Table 4:** Number of microProteins found in the five analyzed monocot species. Small proteins include all proteins smaller than 150 amino acids, including all microProteins. MicroProteins refers to the number of all small proteins identified as a potential microProtein. % Small proteins refer to the relative amount of small proteins compared to the whole genome. % MicroProteins refers to the relative amount of microProteins compared to the number of small proteins

	Barley	Rice	Wheat	Maize	Sorghum
**Total proteins**	35965	43603	105061	39399	34001
**Targets**	26372	29242	90307	32098	26541
**Small proteins**	9593	14361	14754	7301	7460
**MicroProteins**	3810	5451	4482	3790	2534
**Single-copy**	1515	3686	697	1537	1474
**Homologous**	2295	1765	3785	2253	1060
**% Small proteins**	27%	33%	14%	19%	22%
**% MicroProteins**	40%	38%	30%	52%	34%

### Potential crop microProteins are related to diverse molecular pathways

**Figure 2:**
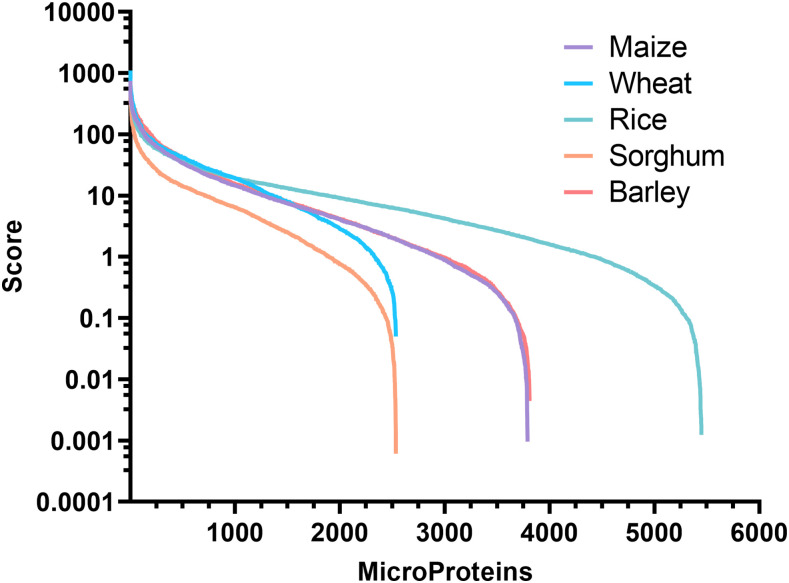
Score distribution of identified candidate microProteins as assigned by miPFinder.

The algorithm identified a large number of candidate microProteins in these proteomes, whose scores follow a power law distribution (Figure 2). This means that while the algorithm identified a number of potential microProteins from these large genomes, only a small proportion of them had a high score denoting confidence that the protein acts as a microProtein. At the same time, a majority of the proteins scored low, meaning that while they fulfill the criteria of a microProtein as defined earlier, they are unlikely to function as a potential microProtein. As such, we chose the top 10% of the candidates from each organism for further study as these represent small proteins most likely to act as microProteins. We performed gene ontology analysis on this set of proteins to map their biological processes. Unfortunately, outside of well-studied reference genomes these small proteins were almost completely unannotated, and therefore we had to focus on their identified targets instead. These targets had more complete annotations from which we could infer the role of the microProteins as they are likely to directly regulate their targets.

We took ten highest-scoring targets for each microProtein in the top 10% of identified microProteins and extracted their gene ontology (GO) terms. We analyzed each organism separately using agriGO v2 (Tian *et al.* 2017) and created a combined list of GO terms which we further analyzed with reviGO (Supek *et al.* 2011), sorting the list based on the p-values of the enrichment (Supplementary Table 3). We find that the most significant term is ‘cellular response to stimulus’, followed by ‘RNA biosynthetic/metabolic process’, and ‘catabolic process’. However, this is not unexpected as these are very frequent Gene Ontology terms and cover many different biological functions. Other enriched terms include ‘cell wall organization’, ‘reproductive process’, ‘cellular response to stress’, ‘ubiquitin-dependent protein catabolic process’, and ‘transcription’, while less-enriched terms include ‘methylation’, ‘response to light stimulus’ and ‘regulation of hormone levels’. As almost all known microProteins are related to transcription factors, it is not surprising to see that ‘transcription’ and ‘gene expression’ are enriched in the GO analysis. The term cellular response to stress implies that some microProteins may be controlling biotic and abiotic responses to stress in plants. This also ties in with the ubiquitin-dependent protein catabolic process, as stress can induce protein misfolding and/or aggregation, warranting the need for the cells to remove erroneous proteins. This process could be controlled by microProteins which are able to quickly turn these processes on and off. Equally, ‘reproductive process’ is a broad term but microProteins which affect flower development (Xu *et al.* 2019) (and therefore the sexual reproduction of a plant) are known, so other microProteins which fine tune this process possibly exist as well. Finally, response to light stimulus is another category which is already controlled by known microProteins (Hyun and Lee 2006) and is especially amenable to microProtein control as light conditions for plants (*e.g.*, direct light *vs.* shade) can change very quickly, requiring a rapidly controlled response from the plant. On the other hand, there are currently no publications that demonstrate microProteins being involved in methylation and response to hormone levels. These processes, however, could be under microProtein control as both protein methylation and hormone signaling require fine temporal control by enzymes and receptors, respectively.

### Analysis of rice microProteins reveals their role in plant development and stress response

In order to test the predictions of the new miPFinder algorithm, we compared the predicted microProteins and their targets against a recent rice RNA expression dataset across 10 tissues. As one of our aims was to identify microProteins that have a fundamental biological role in plant development, we first took all rice microProteins as identified by miPFinder v2.0 and searched for their homologs in all of the other four analyzed species (barley, wheat, maize or sorghum). We reasoned that any protein that is conserved in at least three of the four other species represents a potential microProtein that has a shared essential role across monocots and perhaps beyond. From a total of 5448 predicted rice microProteins we found 422 microProteins that have an identified homolog in at least three of the other analyzed species. We then associated each of these potential microProteins with their highest-scoring target for further analysis. Due to the requirements of the downstream software we had to convert the UniProt accession names into Ensembl transcript identifiers. For a number of microProtein-target pairs one or the other did not have a corresponding transcript identifier and thus had to be excluded from the analysis, leaving us with 347 potential microProtein-target pairs.

We mapped all previously characterized gene information from funRiceGenes database (Yao *et al.* 2018) to the microProtein targets and extracted their biological role keywords, which were divided into individual words and phrases (Table 5), and found 28 and 24 previously characterized targets and microProteins, respectively. We found targets mostly involved in abiotic stress response such a salt or drought, as well as a few targets that control flowering time and yield. The individual keywords were similar to the annotated phrases, with root and growth annotation being the most prevalent, followed by auxin, stress and development. Reassuringly, we found homologs of known microProteins and their targets among the annotated proteins. The target Os12g41860, which is annotated as a member of the class III HD-ZIP family, is co-expressed with the microProtein Os04g33560, which is homologous to LITTLE ZIPPER 3 microProtein. Equally, Os07g49460 which contains a CCT (CONSTANS, CO-like, and TOC1) domain has been linked to flowering (Koo *et al.* 2013). In *A. thaliana*, CONSTANS is a known target of microProtein regulation which together with the microProteins represses flowering (Graeff *et al.* 2016), and so it likely that this target is under similar control by a yet uncharacterized microProtein. This rice target belongs to the ARR-like protein family which is a two-component response regulator. It is interesting to note that the target does not contain the conserved catalytic aspartic acid residue necessary for the phosphorelay system, having a glutamic acid residue instead (D114E). The microProtein, which lacks the CCT domain, has the conserved aspartic acid present. It has been shown for *A. thaliana* ARR18 that the wild-type protein containing the conserved aspartate (ARR18^WT^) is able to homodimerize with a constitutive gain-of-function mutant where the aspartic acid was mutated to a glutamic acid (ARR18^D70E^), but not the case of a constitutive loss-of-function mutant containing asparagine (ARR18^D70N^). Importantly, this gain-of-function ARR18^D70E^ mutant was able to activate transcription of a reporter gene (Veerabagu *et al.* 2012). It is possible that in rice the target, due to its aspartic to glutamic acid mutation, is constitutively active and that the dimerization with the microProtein provides modulation of the transcriptional activation activity by sequestering these active homodimers into inactive heterodimers. Crucially, while they are homologous to known *A. thaliana* microProteins, they nevertheless represent novel microProtein-target pairs that may be controlling plant biological processes.

**Table 5:** Frequency of biological role keywords occurring in the identified microProtein targets. Each target may have had more than one keyword associated with it. Number in parentheses after each term indicates the frequency of occurrence

Target keywords (n = 124)	Target phrases (n = 64)
Root (7)	Transcription factor (5)
Growth (6)	Salt stress (4)
Auxin (5)	Abscisic acid (3)
Stress (5)	Flowering time (2)
Development (5)	Heading date (2)
Shoot (4)	Grain yield (2)
Grain (4)	Auxin response (2)
Drought (4)	Drought stress (2)
Salt (4)	Stress tolerance (2)
Seedling (3)	Cold stress (2)

Finally, we analyzed which microProtein-target pairs were co-expressed on mRNA level. While this does not consider post-translational regulation of the protein, it is a useful tool for identifying genes with a shared function. We analyzed the gene expression correlation patterns for the final list of 347 microProtein-target pairs across 10 anatomical tissues. We found 56 microProtein-target pairs with significant positive or negative expression correlation (-0.7 < r > 0.7 and p-value < 0.01; Supplementary Table 4). This means that out of 347 identified microProtein-target pairs, around 16% are co-regulated with their highest-scoring target as identified by miPFinder, indicating that it might be their biological interaction partner. Overall, this shows that the identified microProtein-target pairs regulate plant processes such as transcriptional control, stress response and reproduction, and that the expression of many of the identified targets are significantly correlated at the RNA level.

## Discussion

The post-translational proteome is an attractive target in biotechnological research as it allows researchers to target specific protein states (*e.g.*, post-translational modifications, conformations, splice variants) (Cattaneo and Chirichella 2019). MicroProteins can be harnessed as biotechnological tools as they have specifically evolved to control protein-protein complexes. Furthermore, the recent use of synthetic microProteins for post-translational regulation of plant proteins in Arabidopsis and rice has demonstrated that these small proteins are not limited to transcription factors (Eguen *et al.* 2015; Graeff *et al.* 2016; Bhati *et al.* 2018; Dolde *et al.*, 2018; Eguen *et al.*, 2020). With advances in next-generation sequencing and genome annotation quality, data from novel organisms that may contain microProteins is becoming more prevalent. For example, small open reading frames (smORFs) which are starting to receive more attention (Su *et al.* 2013), are a potentially huge untapped source of *trans*-microProteins. This suggests that many biological pathways may be regulated by undiscovered microProteins. Such *trans*-microProteins could be utilized in biotechnological research such as crop bioengineering. It has also opened up new opportunities to explore the function of new genes and pathways involved in adaptation to changing environments (Varshney, Tuberosa, and Tardieu 2018). There are examples where involvement of mRNA variants producing functional truncated proteins have recently been described in crop plants, such as the alternatively spliced form of the maize ATHB17 protein. A smaller ATHB17 isoform functions as microProtein and positively affects the expression of genes by suppressing the repressor activity of the full-length ATHB17 protein. Overexpression of the ATHB17 microProtein variant resulted in increased kernel weight (Cantu *et al.* 2013; Rice *et al.* 2014). By regulating specific targets, microProteins can provide attractive means to alter traits of interest for crop improvement. More examples can be found in genomic data from alternative transcriptome sequencing of stress and other biological conditions (Barbazuk, Fu, and McGinnis 2008; Panahi *et al.* 2015; Liu *et al.* 2018; Zhang *et al.* 2017).

However, the accurate detection of microProteins from genomic data remains a challenge due to the biochemical and biophysical similarity to other small proteins. Our lab has previously developed the first ever algorithm for this purpose, called miPFinder, and utilized it to find new microProteins in the *A. thaliana* genome (Straub and Wenkel 2017). We rewrote and updated the algorithm to improve its accuracy and reduce its reliance on annotation databases and demonstrate that the new version is able to identify all known *A. thaliana* microProteins from the set of small proteins. The algorithm is also able to correctly predict the known targets of existing microProteins, although in some cases the predicted target is a homolog of the true interacting partner, and there are cases where the algorithm disagrees with other computationally predicted sources. However, this does not necessarily mean that the identified proteins are incorrect, as they may still be relevant biological binding partners.

In order to find novel microProtein targets for future crop improvement, we used miPFinder to analyze the genomes of five monocotyledonous crop species - wheat, barley, rice, sorghum and maize. We found that approximately 9% of all proteins were identified as potential microProteins. While this represents a large fraction of the overall proteins, only a small percentage (10–20%) of potential microProteins are scored highly, indicating their likelihood of functioning as a real microProtein. It is noticeable that the abundance of microProteins is independent of genome size. Irrespective of having the largest genome size, wheat does not contain the highest number of microProtein candidates. This could be partially attributed to the less efficient genome assembly and low confidence structural protein annotation from the complex allohexaploid wheat genome (Brenchley *et al.* 2012). An alternative argument for this observation could also be that the highly repetitive genome organization does not influence microProtein evolution.

Looking at the highest scoring microProteins in all genomes as well as those identified to be co-regulated in rice, we found that these putative microProtein targets are most commonly involved in the ‘control of transcription’ and ‘stress response’, as well as ‘hormone signaling’, ‘methylation’, and ‘light level response’. In recent years, more studies have been emerging that demonstrate the importance of microProteins in regulating these biological processes. In a recent study, a maize F-box protein called COI1 was found to generate a novel alternative transcript in response to drought stress (Thatcher *et al.* 2016). This truncated *COI1* mRNA consists of only the F-box domain but has lost the C-terminal leucine rich repeat domain. Such a truncated F-box protein could potentially function as a “decoy”, as defined by Lee and colleagues. The recent study of these decoy F-box protein networks involved in circadian clock function strongly endorses the importance of such truncated F-Box microProteins in the regulation of plant development in response to environmental stimuli (Lee *et al.* 2018). Equally, the inferred regulation of protein function and gene expression by methylation and other similar transient modifications are known to regulate development and stress adaptation in crops (Gardiner *et al.* 2018; Wang *et al.* 2011).

In summary, our study extends the understanding of microProteins in cereal crops and shows that crop genomes are rich in high-confidence microProteins. The potential microProteins in monocots cover the unexplored regulation mechanisms of stress and growth pathways, and provide a possible molecular link between biological and environmental processes. The rapid development in genome-engineering tools such as CRISPR-Cas9 will allow us to uncouple microProtein-regulated pathways by generating loss-of-function mutants; such plants can then be studied for their phenotypes under respective biological condition which can aid breeding strategies to enhance desirable traits in crops. As a future perspective it would be of interest to investigate the relationship between the spatio-temporal microProtein expression levels and the resulting phenotypes to better understand how microProteins are contributing to various aspects of plant development in a tissue and environmental-condition specific manner. Such investigations will challenge and revolutionize our current understanding of trait regulation and crop improvement.
